# University Staff’s Perceptions of Community College Transfer Students’ Transition Experiences Within a “2+2” Pathway in an Asian Educational Context

**DOI:** 10.3389/fpsyg.2022.808179

**Published:** 2022-03-04

**Authors:** Shirley Siu Yin Ching, Wilson Yeung Yuk Kwok, Jeremy Tzi Dong Ng, Lillian Weiwei Zhang, Ceci Sze Wing Ho, Kin Cheung

**Affiliations:** School of Nursing, The Hong Kong Polytechnic University, Kowloon, Hong Kong SAR, China

**Keywords:** transfer students, qualitative study, staff perception, higher education, mental health, transition and adaptation

## Abstract

Various countries have alternative pathway policies for 2-year community college graduates to articulate to 2-year university study, forming a “2+2” pathway. However, few studies have explored university staff members’ perceptions of this “2+2” transfer pathway and their understanding of transfer students’ (TSs) transition experiences. This descriptive qualitative study addressed this research gap. Forty-two academic and supporting staff participated in the focus group interviews. Specifically, the study explored the assets and challenges of the “2+2” pathway from the university staff perspective in Hong Kong. The articulation pathway and TSs are highly recognized for their prior learning, academic performances, and the value of the second chance. However, while the university staff were sympathetic to the challenges filling these transfer pathways, their offering of help was limited by government funding and policies restrictions. It is recommended that policies should be established at government and university levels to recognize and tackle TSs’ unique needs to alleviate their heavy workloads through better articulation between community college and university studies. Improving articulation will allow TSs time for social involvement in university life and thus enhance their mental well-being.

## Introduction

In recent decades, there has been a growing volume of literature pertaining to community college transfer students’ (TSs) experiences of transitioning from community colleges to universities. The majority of existing studies focused on their learning experiences and outcomes, such as academic performances (Wang, 2009; Cheung et al., 2020a) and adjustment needs (Owens, 2010; Cheung et al., 2020b; Ching et al., 2021). These previous studies investigating the student transition experience have emphasized the important role of staff, departmental, and institutional support in the transition and learning experience, and the success of community college TSs (Zhang et al., 2018; Howerton et al., 2019). Although some extant studies have explored the pre-transfer preparatory support given by community college staff and the institutions themselves (O’Donnell et al., 2018), there is a lack of coverage of the viewpoints of staff in the receiving institutions (i.e., universities), despite this being one major stakeholder groups in higher education. In other words, relatively little attention has been paid to understanding this non-traditional student population from the perspective of the university staff. As pointed out by Landeen et al. (2017), having a better understanding of this perspective could inspire a higher level of institutional and departmental policies to support and facilitate students’ transfer and transition experiences.

### An Alternative Pathway and Transfer Students’

In addition to the direct admission route, the higher education systems in most countries, western and eastern alike, have alternative pathway policies for secondary school graduates to articulate to university study after obtaining a community college degree. In the United States, a typical “2+2” pathway includes 2 years in community college and 2 years in university (Remington et al., 2013). This is similar to the articulation pathways in Asian countries including China, Malaysia, and Thailand (Lee, 2014; Gao et al., 2016). Globally, the populations of community college TSs in higher education institutions have been growing in recent decades (Archambault, 2015; Wyner et al., 2016).

The “2+2” pathways provide an alternative in a competitive education system. In the study context of Hong Kong, only about one-third of the secondary school students who sit for the Hong Kong Diploma of Secondary Education Examination (HKDSE) can be admitted directly into local University Grants Committee (UGC) funded universities. In the academic year 2019/20, there were 52,416 HKDSE candidates competing for 18,362 (35.0%) first-year-first-degree (i.e., direct entry) places distributed across eight publicly funded universities [Hong Kong Examinations and Assessment Authority, 2020; University Grants Committee (UGC), 2020]. There were 28,200 sub-degree (i.e., community college) intake places from more than 300 programs offered by 26 local sub-degree institutions (i.e., community colleges) in the 2019/20 academic year [Concourse on Self-financing Post-secondary Education (CSPE), 2020]. For the two-thirds of the candidates who cannot meet the competitive admission requirements, attending a community college with a better graduation score is one alternative pathway into a publicly funded university; the other option would be to retake the public examination. Graduates of 2-year local accredited sub-degree programs (i.e., associate degree or higher diploma) in relevant disciplines from institutions in Hong Kong or equivalent, can enter directly to Year 3 of selected bachelor’s programs, forming a “2+2” pathway. As there is no gap year with the articulation, thus, community college programs are popular. Even though the admission requirements of these programs are relatively lenient, the articulation of college-to-university is also competitive. There are only 5,000 places for “2+2” pathway intakes in eight publicly funded universities [Concourse on Self-financing Post-secondary Education (CSPE), 2019]. Although overseas universities or local self-financing universities might offer more places, local candidates prefer to attending publicly funded universities.

### Transition and Learning Experience of Transfer Students’

Despite the transferability, previous findings have suggested that community college TSs, as a group of non-traditional, disadvantaged higher-education students from academic, social, and psychological perspectives (Cheung et al., 2020a,d; Ching et al., 2021; Lau et al., 2022). Some authors have argued specifically that the transfer pathway is insufficiently structured and supported (Jaggars and Fletcher, 2014; Baker, 2016). Numerous studies of the learning experiences of community college TSs have focused on the academic, social, and psychological aspects of these students’ needs. Issues related to the academic aspect are the common phenomenon of transfer shock, referring to a drop in post-transfer GPA after arriving in university (Cheung et al., 2020a; Ching et al., 2021), higher attrition rates (Aulck and West, 2017), and heavier study loads than those of other groups of students (Xu et al., 2018; Cheung et al., 2020a,c; Ching et al., 2020b, 2021). Other research has identified TSs’ exposure to additional academic distress (Mehr and Daltry, 2016; Cheung et al., 2020c), higher levels of depression, anxiety and stress than experienced by other groups of students (Beiter et al., 2015; Cheung et al., 2020d), insufficient integration with their peers and inactive engagement in social activities (Archambault, 2015; Xu et al., 2018; Cheung et al., 2020b; Ching et al., 2021). Given the clear portrayal of undesirable overall learning experiences of TSs; however, university managements and administrators have still been criticized for lacking an understanding of their characteristics and needs (Tobolowsky and Cox, 2012; Nuñez and Yoshimi, 2017). University staff are the ones who either make or implement decisions regarding students’ academic and non-academic affairs, so it is important to explore their perceptions of this “2+2” alternative pathway, particularly because the transition experiences of these non-traditional students are often neglected at the institutional level.

Previous studies of students’ perceptions of their personal and social experiences have revealed a spate of difficulties. In a qualitative study conducted in the United States, it was found that TSs experienced stigma on campus after their enrollment in university. They felt uncertain about their academic abilities to succeed in university and were afraid of the stereotypes of their sociodemographic disadvantages (Shaw et al., 2019). Studies in Asian contexts such as Hong Kong have indicated that TSs felt inferior and less competent than direct entrants (DEs) who were admitted directly to university based on their university entrance examination scores. These feelings of inferiority occurred even though the TSs’ graduation grade point averages (GPA) were higher than those of their DE peers (Cheung et al., 2020a; Ching et al., 2020b, 2021). It is not clear if university staff have similar perceptions of the transfer pathway and thereby recognize the possible challenges of TSs. However, worldwide, research concerning students’ social relationships and interactions from the perspective of university staff is relatively insufficient. As a matter of fact, teacher support is a crucial factor affecting students’ academic and social integration into university. As suggested by Tinto’s (1975) model of student integration, the most important factor contributing to students’ persistence in studies is their integration into the academic and social systems of the institution, especially for TSs (Townsend and Wilson, 2009). Furthermore, there was a discrepancy found between students’ and teachers’ perceptions; teachers experienced the support given to students more positively than that perceived by the students (Asikainen et al., 2018). In more controversial cases, as reported by Lähteenoja and Pirttilä-Backman (2005) in Finland, some university teachers expressed negative attitudes toward student integration, perceiving it as unnecessary.

### Institutional Support for Transfer Students

Success for community college TSs appears to be linked directly to the development of quality transition programs in institutions and individual departments (Howerton et al., 2019). In order to create environments for better transfer and transition experiences, institutional and departmental support are vital to TSs’ academic and social involvement and integration at university (Baker, 2016; Bowker, 2021). In particular, academic staff play a significant role in TSs’ successful degree completion by developing engaging courses, helping them develop rigorous strategic plans to reach their academic goals, and establishing meaningful relationships with them (Howerton et al., 2019). Furthermore, statistically significant positive relationships have been identified between the frequencies of interactions between TSs and faculty, and their experience with academic (Lopez and Jones, 2017) and social (Laanan et al., 2010) adjustment, and coping with problems (Moser, 2013). These findings corroborate the benefits of staff support for TSs. Furthermore, Zhang et al. (2018) found that the level of institutional support and academic related interactions with academic staff were associated significantly with the likelihood of TSs being satisfied with their university experiences. Since academic and support staff at the universities are among the major stakeholders whose decisions and actions can facilitate or hamper the transfer process, it is essential to explore their perceptions of institutional resources and actions in order to ensure that optimal levels of support are directed to community college TSs in university.

### Studies on Transfer Students in Asian Educational Context

While the aforementioned studies were mainly conducted in western cultures [e.g., the United States (Tobolowsky and Cox, 2012), Finland (Lähteenoja and Pirttilä-Backman, 2005; Asikainen et al., 2018), Canada (O’Donnell et al., 2018; Bowker, 2021)], community college transfer also prevails in Asian countries and regions, including Hong Kong, mainland China, Malaysia, and Thailand (Gao et al., 2016; Cheung et al., 2020a). In Asian societies, academic qualifications and employability are often emphasized because of social norms (Tien and Wang, 2017). In particular, it is increasingly common for degree aspirants in Greater China to earn post-secondary credentials before commencing university studies (Peng and Hui, 2011). On a related note, in at least one Asian educational context, Hong Kong, students’ degree aspirations are getting stronger (Wan, 2006). Post-secondary education has undergone a flourishing growth since 2000, with the Government policy of enhancing Hong Kong’s global competitiveness and developing a knowledge-based economy by democratizing higher education to a wider population [Hong Kong Special Administrative Region (HKSAR) Education Commission, 2000]. By 2019, in Hong Kong, the number of students in full-time post-secondary education programs had increased to 179,700 [Concourse on Self-financing Post-secondary Education (CSPE), 2020] from 23,758 in 2000 [University Grants Committee (UGC), 2010]. For graduates of sub-degree programs (i.e., associate degrees or higher diplomas) from community colleges, the HKSAR government increased full-time publicly funded undergraduate program places for vertical TSs (Shah and Nair, 2016).

Driven by the influence of traditional Confucian values in modern Chinese societies, it is regarded as a social norm for young adults to pursue higher degrees; this means that students should make all-out-efforts, by any means, to obtain post-secondary qualifications (Lee, 2019). With their prior experiences of failure in university entrance examinations, and thus the need to attend sub-degree programs (i.e., associate degree or higher diploma), community college graduates tend to perceive themselves as “losers” and to be regarded by the public as inferior (Lee, 2019; Wong, 2020). This has, therefore, contributed to community college graduates having strong aspirations to obtain bachelors’ degrees.

In view of the aforementioned knowledge and research gaps, this study explored the university staff’s perceptions of the community college transfer pathway and TSs’ learning experiences in an Asian educational context. The two research questions (RQ) were:

RQ1: How do university staff perceive community college transfer as a pathway to university study in Hong Kong?

RQ2: How do university staff perceive/understand the learning experiences and challenges encountered by community college TSs in Hong Kong?

## Materials and Methods

A qualitative approach was adopted to explore the perceptions and experiences of university staff in supporting TSs before, during and after their transfer. As this understanding of staff perceptions has been limited in the literature, a qualitative approach of interviewing participants with relevant experience was a good fit for the purpose of the current study (Henwood and Pidgeon, 1992).

### Research Context

The university under investigation hosted about 35% of all TSs in Hong Kong in the past 5 years [Concourse on Self-financing Post-secondary Education (CSPE), 2020], as one of the major publicly funded institutions providing TSs with articulation to baccalaureate education from community college studies. It should be noted that, in Hong Kong, there is no independent TS-exclusive institutional support, such as a transfer center, available. Instead, the support from the institution is available to all students, regardless of their admission routes.

### Participants

Participants (i.e., university staff) were recruited from the above-mentioned university using purposive and snowball sampling. The inclusion criteria were (1) employed academic or support staff in the university; (2) experienced in teaching and/or supporting TSs; and (3) able to communicate in English or Chinese. Internal invitations were sent to target faculties and departments through internal email. Interviewees who attended the interviews were also asked to invite colleagues to participate in the study. Overall, 42 university staff members (52.4% female) were recruited from all eight academic faculties/schools, and six supporting units (e.g., Student Affairs Office). Table 1 indicates the information of 42 participants. The interviewees were assured that their personal identities would be kept strictly confidential. They had the right to withdraw at any point of the study without any consequences. They were invited to sign a consent form to show their awareness of above information and agreement to participate in the study. Their participation was voluntary and no incentive was offered to them. Institutional ethical approval was obtained (HSEARS20180104005-01).

**TABLE 1:** Information about participants.

Faculty/Unit	Number of interviewees (*n* = 42)	Gender
**Academic staff**		
Faculty of Applied Science and Textiles	1	Not provided to prevent the possibility of the identification of the participant
Faculty of Business	2	2 Males
Faculty of Construction and Environment	4	1 Female 3 Males
Faculty of Engineering	4	4 Males
Faculty of Health and Social Sciences	8	5 Females 3 Males
Faculty of Humanities	4	2 Females 2 Males
School of Hotel and Tourism Management	4	2 Females 2 Males
School of Design	1	Not provided to prevent the possibility of the identification of the participant
**Supporting staff**		
Office of General University Requirements	6	4 Females 2 Male
Student Affairs Office	4	3 Females 1 Male
Service-Learning and Leadership Office	3	3 Females
Library	1	Not provided to prevent the possibility of the identification of the participant
Total	42	22 Females (52.4%) 20 Males (47.6%)

### Data Collection

A total of 33 sessions of interviews was conducted, consisting of five semi-structured focus groups and 28 individual interviews, according to the availability of the 42 university staff. The interviews were conducted in quiet rooms in the university, and each lasted about 30–90 min. The interviewees were free to share their perceptions and experiences about TSs in the university. To answer the research questions, an interview guide was developed based on the literature review, focusing on the participants’ perceptions of the community college transfer pathway (RQ1), and the learning experiences of community college TSs (RQ2).

The interview started with a general question, “Can you tell me about your experiences with TSs in the university?”. Based on the responses, the interviewer asked probing questions to get more information and clarify the conceptualized message. The interview guide also covered the scope of the topics, reminding the interviewer to conduct a broad and in-depth interview. For instance, the perceptions of university staff toward TSs’ (1) articulation pathways; (2) transition and adjustment processes; (3) learning and social experiences; and (4) the needs and challenges encountered by TSs; and (5) the support given to TSs in the university. All the focus groups and individual interviews were audio-recorded by digital recorders. The data collection was continued until data saturation was achieved, when no more new information emerged or the scope of experiences was being covered repetitively.

The interviews were conducted in either English or Cantonese. During the individual interviews, the interviewer, who is a researcher with experience of qualitative studies, asked questions and explored the participants’ perceptions. During the focus groups, one interviewer raised questions and facilitated discussions among the participants and a research assistant took brief notes so that follow-up questions could be asked to elicit ideas/hunches/thoughts (Krueger and Casey, 2001). This study embraced and focused primarily on an epistemological standpoint to explore the multidimensional contexts of university staff’s perceptions of community college “2+2” transfer pathways and their understanding about transfer students’ (TSs) transition experiences. The combination of individual interviews and focus groups enhanced the richness of the data and broadened the understanding of the phenomena of interest (Lambert and Loiselle, 2008; Carter et al., 2014). The individual interviews drew out personal and sensitive opinions about the transfer pathways and TSs while the focus groups obtained data resulting from the interactive discussions among the participants to increase the depth of the research questions.

### Data Analysis

Staff members’ experiences were multifaceted and sensitive. In this exploratory study, qualitative content analysis was adopted for the simple reporting of common issues mentioned (Vaismoradi et al., 2013). Specifically, a manifest approach was followed; the researcher focused on describing the directly observable and obvious elements without assigning a meaning at the time of coding (Graneheim and Lundman, 2004). Throughout the inductive inquiry process, interpretation was kept to a minimum level to ensure the trustworthiness of the data analysis and the consistency between different researchers (Graneheim and Lundman, 2004; Malterud et al., 2016). Bracketing was used to reflect the participants’ personal experiences and knowledge to ensure the findings of the study were shaped only by the respondents, without any researcher bias, personal values or judgmental attitudes (Polit, 2018).

As mentioned, two types of data collection approaches were adopted. However, the findings demonstrated nuanced differences in the participants’ understanding of the transfer pathway and perceptions of TSs that were not anticipated initially. The data collected from these two approaches were combined for data analysis. All interviews were recorded and then transcribed verbatim in Chinese/English to maintain the literal meaning, then imported into NVivo Pro 12 for subsequent analysis. At the beginning of the data analysis, the researchers read the interview notes, and transcribed the texts of a few interviews to get a full understanding of them as an “immersing” experience. Then the meaning units presenting the most detailed aspects of the participants’ experiences were condensed into codes. The codes were collated into sub-categories and then categories based on their similarities or differences (Vaismoradi et al., 2013). They discussed the codes and categories for consensus before analyzing the subsequent interviews. Four research assistants worked on the data analysis collaboratively, with monthly meetings to discuss the main findings to ensure the consistency and trustworthiness.

## Results

Two main categories and their sub-categories were identified in our analysis: (1) assets of the “2+2” pathway: TSs’ prior learning in community college, instrumental learning approaches, and valuing of the second chance for TSs; and (2) challenges to learning under the “2+2” pathway: a hard-earned second chance, constrained by the government funding, and sympathetic but unable to help the TSs.

### Category 1: Assets of the “2+2” Pathway

The participants expressed a belief in the 2+2 articulation pathway, which provides an alternative admission route toward baccalaureate degrees after graduating from the community college. The participants believed that, despite this baccalaureate admission route being indirect, the prior learning in the community college nourishes and equips the TSs to reach the standard performance of the average university student. The staff also suggested that the TSs had demonstrated highly motivated and self-directed learning in their studies, adopting instrumental learning approaches, with timely graduation as the ultimate goal. More specifically, the participants had the view the TSs value and treasure this second chance of getting into a university after failure in the city-wide university entrance examinations, and thus strive to do their best after the articulation. These perceived strengths of the community college transfer and TSs show the staff’s confidence in the 2+2 pathway being a circuitous but viable route to baccalaureate degree studies.

#### Transfer Students’ Prior Learning in Community College

The interviewees commented that the curriculum structures of the community college programs were aligned with those of baccalaureate degree programs in university. They remarked that the 2-year training in the community college is comparable to the first 2 years of a typical 4-year university program, providing a solid foundation of knowledge in specialized disciplines and practical vocational skills for the TSs. Coupled with this, the participants suggested that the TSs usually articulate to degree programs in discipline areas that interest them the most and that are related to their community college studies. Therefore, they believed the TSs were able to catch up smoothly after articulation, which could be interpreted as showing confidence in the articulation and their overall recognition of the transfer pathway.

*It (the ability to learn key concepts) depends. TSs’ 2-year higher diploma or associate degree studies have already developed the understanding of basic concepts of a certain discipline or profession. Their ability to understand is not bad if the field of study at the university is relevant to their prior learning. The adaptation in academic aspects was smooth as they caught up quickly (29A, academic staff).*

*Actually, we only select TSs from a very small number of relevant associate programs, so their study backgrounds are controlled by the professional board. The skills they have learned in the higher diploma even from other institutions using different modes of delivery, I believe are quite similar if not identical (35A, academic staff).*

The participants understood that competition is high in the community colleges, as students have to achieve outstanding academic performances in order to earn the chance to articulate to baccalaureate studies. They explained that applicants who obtained GPAs lower than 3.2 out of 4.0 would not be considered by the university during the articulation process. They viewed this competitiveness arising from the transfer pathway as playing a gatekeeper role to select the most suitable and competent students. Thus, they considered the TSs who succeeded in articulation to be the cream from the community college, with a guaranteed quality matching the standards of the university.

*The minimum GPA requirement for the TSs admission is above 3.2. The applicants who achieve less than 3.2 in GPA will not be considered for interview. Thus, the students who articulate to our university had performed very well academically in their community colleges (17A, academic staff).*

*I think most of them are hard-working, with good learning attitudes because, as I said, if they are promoted from the higher diploma program, they have had to compete for the degree places, so they need to be hard-working and get high GPAs in order to be promoted to the TSs program (07A, supporting staff).*

The staff said they were aware of a common misunderstanding among outsiders that TSs who had failed once before would not perform as well as DEs. Some participants observed that TSs usually graduate with higher award GPAs than the DEs. In general, a large portion of TSs obtained GPAs higher than 3, which was seen as “very good.” One academic staff member even stated that the first honors degree holders in the last few years in his department were all TSs.

*That’s why I’ve just highlighted that TSs being admitted through the turbulent route do not equate to poor academic results. Some may suggest that this group of students who failed in the public examination before would not perform as well as the DEs. This, in fact, was contradicted. These groups of students perform very well academically. In the last few years, the first-class honors students were the TSs, while there were none from DEs (29A, academic staff).*

#### Instrumental Learning Approach

The participants had observed that TSs tend to be more mature, having somewhat well-planned life orientation in both their studies and career trajectories, and are heavily goal-oriented. In particular, they strive for high award GPAs and to graduate on time. They were seen as taking an instrumental and pragmatic approach to their studies, making every effort to achieve high levels of academic performance. The interviewees recalled that TSs are keen to stay attentive in class, grabbing the details about assignments, grading requirements, and tips for getting high scores at the beginning of the course, so they could make study plans early on.

*They asked for details about guidelines, assessment rubrics, and timelines, and even asked about plans which were not yet published this year (19A2, Supporting staff).*

*If comparing these two groups of students (TSs and DEs), I still find, generally speaking, that the TSs tend to be more mature, motivated, engaging and more self-responsible, and more interested in their career planning (31A, academic staff).*

In class, they found that the TSs demonstrate better attentiveness, more active involvement and engagement. One participant recounted that the TSs had even approached him before the semester started.

*These students have approached us more frequently than the bachelor (DEs). They are much more motivated, ask more questions during classes (24A, academic staff).*

*When I was handling their enquiries, I found that the TSs were more mature in communication, and they even prepared well when making enquiries, in that they had searched for answers, even called our office before the beginning of semester 1 (19A3, supporting staff).*

The participants suggested that the TSs might have matured from their previous experiences in the highly competitive studies during the 2 years in the community college, and perceived that this might have been beneficial to their adjustment to the first year of university study.

*I think, compared with the DEs, TSs are more mature because when they join the program, they’ve already had 2 years of experience in their associate degree or higher diploma studies. So it’s not too difficult for them to adapt to the university environment I think (21A, academic staff).*

#### Valuing of the Second Chance of Transfer Students

The participants suggested that the TSs have experienced “failure” in their university entrance examinations, so seize and treasure this “second chance” of pursuing the baccalaureate degree, studying harder despite the hardships in adjusting to the transition and learning process.

*Once they have the chance to further their studies here in order to get a degree, the majority of them study very hard, because at the very beginning they failed to get into this 4-year program, and that’s why they went to the sub-degree institutions to finish their associate degrees or higher-diplomas. This is considered as a second chance to get a degree. And that’s why once admitted to the program, I can see the majority of them are ready to strive very hard to finish a degree here. So that’s why I can see many good results there from this group of students (20A, academic staff).*

*They care more about matters related to their university graduation or degree pursuit, or fulfilling requirements, and they have very specific agendas about doing this (08A2, supporting staff).*

The participants also held the view that TSs cannot afford any more “failures”, such as any obstacles and mistakes that would harm their academic performances or even lead to delays in graduation. From the participants’ experiences, TSs are easily stressed and even confront their teachers when they encounter the common phenomenon of transfer shock, i.e., a drop of GPA in the first semester of university study.

*I feel that they’re more stressed than DEs students*,… *every semester, they’re taking the maximum credit load. They cannot afford wrong subject registrations, because they have placements and other modules to catch up with. So they became more nervous about the subject registration and their results, because they don’t really have time or quotas to retake the same modules again (07A2, supporting staff).*

*I know some students did really well in their higher diplomas or associate degrees, but when they get into our university, and then after the first semester, they will visit me because they are disappointed [with the difference between their sub-degree and current GPAs] (08A3, supporting staff).*

### Category 2: Challenges to Learning Under the “2+2” Pathway

When asked about TSs’ learning experiences in university, the 2+2 pathway was depicted by the participants as a route packed full of challenges. As repeatedly emphasized, this pathway serves as a second chance for TSs to pursue the baccalaureate studies, as an achievement after the 2 years of hardship in the community college. Nonetheless, as a hard-earned second chance, this transition and pathway are full of obstacles, namely heavier study loads and greater mental health burdens. These were acknowledged by the participants as consequences of both limited credit transfer and the mandatory 2-year curriculum; they expressed sympathy toward TSs but also helplessness to support them due to structural issues such as restrictions from government funding.

#### Hard-Earned Second Chance

The participants indicated that the TSs have heavier study loads than DEs, leading to less involvement in university campus life and thus less participation in extra-curricular activities. This was noted as the cost of this second chance. Even during the summer semester, from the participants’ observations, TSs have to attend classes to fulfill curriculum requirements, while the DEs can usually enjoy a semester break. One participant shared his communication with the TSs about their heavy study loads:

*When they come to me, it is all about the workload (20A, academic staff).*

*Because, as I said, their study load is really heavy*,…. *they need to be more self-directed, they need to have good planning, and they need to take up more than seven subjects sometimes in one semester. Also, during the summer semester, they have to do their placements, so actually they will be fully occupied in the 2-year university study] (07A1, supporting staff).*

In addition, some participants pointed out that the university discourages TSs from joining exchange program, which is considered as one of the must-do activities in the university. They are discouraged because it will lengthen the duration of their supposedly 2-year studies.

*They don’t have enough time to enjoy any other learning and university experiences like exchange or others like minor studies.*… *so I think the main difference is they do not have much university experience when compared with the 4-year degree students (07A1, supporting staff).*

*Overseas exchange is another matter because if you have read some guidelines in general, the university does not encourage TSs to have exchanges because that will obviously lengthen their study period. The university would not like to see too many students extend their study periods (35A, academic staff).*

#### Constrained by the Funding Resources

The government only provides funding to the universities for the 2 years of baccalaureate studies for TSs, in other words, the final 2 years of the 2+2 pathway. An extension of this duration, for example, an additional semester, would be considered over-enrollment, meaning that no government resources would be provided. Thus, TS’ studies should always be completed within 2 years.

*It’s difficult to change the program pattern, because the government only gives us 2 years’ worth of funding, so unless the government can increase it to another year, then we can’t actually extend the program to 3 years. So they’ve got a lot of subjects to cram in, because it’s more for the structural issue, policy issue than what we can do (05A, academic staff).*

*So, we only get 2 years’ resources, and what can you do within the 2 years? So, that’s a lot of requirements we have to address. As I mentioned before, all are programs with professional credits, so we have to meet all these criteria, within these criteria, and to develop the curriculum (32A, academic staff).*

According to the participants’ understanding, the extension of the 2-year curriculum is feasible but often not encouraged. They explained that, due to the possibility of compensating for the government funding, the academic decision-makers in the university will give careful consideration to reasons or justifications for delays in graduation before accepting or rejecting them. They mentioned that only a handful of special cases of requests for extension of the study duration were granted.

*So we don’t encourage students to extend their learning. Only in very special situations will we allow them to extend (16A, academic staff).*

*There are policies for graduating on time. The funding council doesn’t encourage extension of the study period. It is a balance between requirements and the designed 2-year study period (35A, academic staff).*

#### Sympathetic but Unable to Help the Transfer Students

The participants were sympathetic to TSs’ challenges and needs, particularly their heavy study loads due to the short and rushed curriculum, coupled with the lack of social involvement in campus life. They agreed that an extension of the curriculum duration would help the students but could not change the fact that this duration is restricted by limited resources:

*You can’t accelerate growth by pulling plants out of the earth. Learning is a process. Like plants, students need time to grow. If they could have one more year, or two more years, it would be better. Then they could get more time to learn practical based content, which is important for their learning (14A, academic staff).*

*I think 4 years, a“2+4” program, would be better. It is a resources issue (10A1, academic staff).*

Some participants said that they were willing to help the TSs, and had tried to get in touch with them to learn more about their challenges and needs. However, there was little they could do as the study loads could not be reduced and they had limited opportunities to interact with TSs outside the classrooms:

*I think they have a lot of credits to study. They have huge loadings. I don’t know how to help them (16A, academic staff).*

*I actually do not have a lot of opportunity to speak to TSs individually in a meeting session because, you know, we are just like an information center, so they just come in, and then we answer questions (08A1, supporting staff).*

Some participants suggested having social gatherings and extra out-of-classroom support to enhance the interaction and relationship.

*If they have already established a relationship with the program leader, then they probably just pop in to his/her office, and then ask the questions (26A, supporting staff).*

*We can also think about collaboration with the department, I think maybe (suggest to) invite the teachers to have some casual gatherings with the students so that they may ask something about their study during the session (07A1, supporting staff).*

## Discussion and Implications

This study has been a pioneer to explore the perceptions of university staff toward community college transfer pathways, and those of the learning experiences of community college TSs. Our findings make a significant contribution to the literature in that they shed further light on the understanding of the transfer pathway and TSs’ learning experiences. On one hand, university staff were confident about the assets of the “2+2” pathway, mainly due to its competitive selection of the strongest community college graduates, and the arduous efforts and diligent attitudes exhibited by TSs. They treasured and valued the second chance despite the hardships. On the other hand, the staff perceived this hard-earned second chance at university study to be full of challenges: TSs have heavy study workloads without time and capacity for social involvement in university campus life within the fixed 2-year curriculum. In spite of being sympathetic, the staff admitted their limited ability to help because of the restrictive 2-year government funding policy. The roles of receiving institutions and their staff will be discussed below. Meanwhile, a synthesis of the concepts of transfer student capital (Laanan et al., 2010) and transfer student success (Fauria and Fuller, 2015) underpins the discussion and implications of the study results in order to make practical recommendations for the benefit of TSs. This study focused on different factors contributing to the success of transfer students, such as academic performances, learning experiences and social involvement.

### Assets of the “2+2” Pathway

The university staff interviewed in our study expressed confidence in the community college transfer pathway as part of the higher education landscape. One of the main reasons was their positive belief that this pathway was designed specifically for stronger students articulating from community colleges. This belief could stem first from the academic qualifications TSs acquire from their community college education (Townsend and Wilson, 2006), and second, from the pre-defined 2-year timeline of the degree pathway for community college TS (Lee, 2014). TSs in western education contexts (e.g., the United States) are demographically diverse in terms of their ages, racial backgrounds, educational backgrounds, and working experiences (Townsend and Wilson, 2006; Mickelson and Laugerman, 2011). However, the majority of their counterparts in Hong Kong, as in most other Asian countries, are local fresh graduates from 2-year sub-baccalaureate programs aspiring for Bachelor’s degrees offered by local universities. As well, the degree attainment rate and attrition rate act as a pivotal institutional performance measure and indication of success of TSs for both universities and community colleges, where community college TSs in Hong Kong are more likely expected to achieve at least a satisfactory academic performance, given their successful attainment of an associate degree or higher diploma prior to transfer (Yuen, 2015; Aulck and West, 2017). Our qualitative findings are in alignment with a previous large-scale quantitative study comparing the academic performances of TSs and DEs in Hong Kong, which reported a statistically significant result that the TSs (*n* = 7,308) had lower attrition rates and there were smaller proportions on academic probation than the DEs (*n* = 14,141) (Cheung et al., 2020a). Consequently, the university staff in our study demonstrated their belief in this pathway to degree attainment and success.

Our study also revealed that the university staff perceived TSs to be heavily goal-oriented toward achieving high GPAs and graduating on time. This is not surprising, as universities have rigorous screening mechanisms that admit only the strongest leavers from community colleges (Xu et al., 2018). This phenomenon is even more rampant in Asian universities positioned as “elite institutions” that tend to take only the best-performing candidates (Kennedy and Lee, 2007; Hannum et al., 2019; p. 74). When studying in community colleges, especially in Asian contexts like Malaysia, Vietnam and Hong Kong (Le, 2013; Ong and Cheong, 2019; Wong, 2020), where articulating to universities is the primary goal, students strive for academic excellence that increases their likelihood of getting places in baccalaureate degree programs (Ching et al., 2020b). Similar to their counterparts in the United States, these degree aspirants are likely to have carried their goal-driven motivation for studying from their previous experiences to their baccalaureate studies (Moser, 2013), except that TSs in the United States might not have attained any pre-tertiary credentials like those in Hong Kong. A recent qualitative study of TSs in Hong Kong (Ching et al., 2021) made an observation consistent with our findings. In that study, the TSs had become accustomed, while in their community colleges, to studying hard to get above-average academic performances, and some may have carried the same learning attitudes and study pattern over to their university studies. Hence, it seems that TSs participate actively only on occasions that will potentially benefit their academic performances, such as only asking questions that are related to assessment. In fact, such a pragmatic approach to teaching and learning has been criticized as both a cause and an effect of instrumentalism in higher education (Karseth, 2008), echoing our findings.

A study suggested that Chinese students are more goal-orientated comparing with students from other countries, they would spend more time in their study and perform well under a highly competitive or stressful academic environments comparing the students coming from different countries (Li, 2017). Another study reported that majority of the Chinese students had high hope for success and high fear of failure (Turner et al., 2021). As suggested by Stankov (2010), the Confucian culture underpinned the desire of academic success among the Chinese students, it put high regard for the academic achievement and failure was deemed as unforgiving. In response to the feeling of guilt and anxiety owing to the failures, Chinese students, affected by the Confucian culture, would appear more critical and attach harsher about themselves. This culture is also applied to the TSs who failed once in the university entrance examination in the study context. They become more hard working and goal-orientated with the failure experience. TSs are not only strived for achieving their own success but also for the honor of their parents. On a positive note, students who have failed once in university entrance exams and are disappointed about their failure are encouraged to seek other second chances to pursue higher education instead of going directly to work. The college-to-university articulation pathway is a popular option. As stated in qualitative studies conducted by Wong (2019) and Ching et al. (2021) exploring the learning experiences of community college students in Hong Kong, during their community college years they tend to reflect on their previously critical educational failure in the public examination and become more mature in thinking about their life goals and developing their own future plans. In this study, the participants (university staff) suggested that these groups of TSs appeared more mature and goal orientated than the direct-entry students, and they interpreted that the difference was a consequence of the experience and the assets of the “2+2” pathway. In the meantime, future study can explore the effects of the failure in a competitive public examination to the personal growth and maturity among the Chinese students and the impact of Confucian culture.

Nevertheless, despite these positive impressions of TSs that were widely agreed upon by the university staff, they actually still need the help and support that ordinary freshmen in a university would receive (Townsend and Wilson, 2006). Migrating from one post-secondary environment (community college) to another (university) leads to TSs’ experiencing “campus culture shock” (Mehr and Daltry, 2016) and “transfer shock” (Cheung et al., 2020a; Ching et al., 2021), with the psychological and environmental adjustments embodied in the transition process making the transfer a “turbulent” experience (Cheung et al., 2020b,c,d; Ching et al., 2020b, 2021). At the same time, TSs generally treasure the “second chance” to pursue university baccalaureate studies. However, their aspirations for academic excellence may bring about a fear of failure, and thereby stress, if their academic performances are outside their own expectations. In fact, it has been identified that TSs commonly experience the problems of “transfer shock” and difficulties in adjusting to the new campus culture, which can lead to an inevitable decline in their post-transfer academic performances, be it temporary or sustained (Townsend and Wilson, 2006; Mehr and Daltry, 2016; Cheung et al., 2020a,b,c; Ching et al., 2021). This suggests that university staff should take proactive action to explore the TSs’ academic and non-academic issues and needs, especially in terms of their transition and adaptation to university studies and campus life (Tobolowsky and Cox, 2012). Moreover, their over-emphasis on academic performance might be induced by the paradigm of outcome-based education in Asian universities for benchmarking with international standards (Kaliannan and Chandran, 2012). Furthermore, increased fear of academic failure not only aggravates TSs’ stress but also discourages them from using deep and organized learning approaches and negatively affecting their self-efficacy (Mørk et al., 2020). Hence, it is important to help TSs maintain all-round development as a prominent aspect of lifelong learning. It was suggested in our interviews that university staff can take on initiatives and actions that maximize and synergize their respective expertise and experiences in order to improve both the academic and the social integration of TSs (Manning et al., 2013). The university staff might consider creating small group consultations targeting the TSs on a department or program basis to facilitate their learning experiences. In spite of the assets of the “2+2” pathway being demonstrated in this study from the university staff perspective, the general image of sub-degree studies and articulation pathways are inferior (Kember, 2010; Yau et al., 2018). This contributes the challenges of poor self-identity and social integration (Cheung et al., 2020a; Ching et al., 2020b, 2021). A qualitative study exploring the local TSs’ learning experience reported that they felt inferior to the DEs who were admitted directly to university even though the TSs’ academic achievements were higher than those of the DEs (Ching et al., 2021). They perceived themselves as incompetent and even as “losers,” and most of them did not participate or interact with other TSs and non-TSs. The findings of this study should be disseminated to the public to improve the social status of the TSs. University staff members’ confidence in the articulation pathway and recognition of attitudes to learning and academic achievement should be promoted widely, to help to bring about a cultural change. For instance, the success stories of TSs could be shared with secondary school students, their parents and teachers to alleviate the effect of labeling the status of the college-to-university articulation pathway as inferior. Instead, the candidates adopting this alternative, a second chance to pursue higher education, who have faced and met the challenges, should be recognized.

### Challenges to Learning Under the “2+2” Pathway

University staff in this study were largely affirmative of TSs’ efforts in their studies and their maturity when compared with DEs. However, these qualities are not sufficient for them to navigate through their academic and social integration (Ishitani and McKitrick, 2010; Cheung et al., 2020b,c). Our study found that university staff did recognize TSs’ challenges of heavy workloads, academic stress, and not being able to participate in extra-curricular activities. In fact, the heavy workloads for TSs have been reported in the United States as well, being another long-standing issue in the transfer literature: the inadequacy of credit transfer systems that force TSs to take up more credits in order to fulfill the baccalaureate requirements or delay their degree completion (Monaghan and Attewell, 2015; Walker et al., 2016). Generally, TSs in western settings have been described as being uninterested in the new campus life and thus less participation in social events, but still participating in events related to their studies, such as out-of-class discussions (Ishitani and McKitrick, 2010; Nuñez and Yoshimi, 2017). They also appear to be more focused than the DEs on completing their baccalaureate degrees (Nuñez and Yoshimi, 2017). Indeed, our results concurred with these observations in western contexts. Nevertheless, our university staff found their TSs faced another unique challenge, which was the need to meet graduation requirements within 2 years. This contrasts with the situation in the United States, where TSs commonly studied for more than 4 years but this was still not treated as deferred graduation (McNeil et al., 2016). Hence, the fixed timeframe also restricted the flexibility of TSs in Hong Kong such that their involvement in the extra-curricular activities and overseas exchange programs was either not feasible or minimal. Furthermore, developing new social networks is another challenge for TSs when they transit to the new campus environment, whereas the DEs already have established social relationships by the time the TSs arrive (Townsend and Wilson, 2006; Mehr and Daltry, 2016).

In view of these difficulties, the university staff in this study expressed their sympathy toward TSs but felt helpless about resolving the situation. Due to the possible lack of communication between academic departments and student support units (e.g., Student Affairs Offices) beyond administrative and routine matters (Abubakar and Danjeka, 2016), academic staff with heavy research and teaching loads might not have the capacity to deepen their awareness of the characteristics and needs of their students, particularly non-traditional students like TSs (Scott and Lewis, 2011; Kwiek and Antonowicz, 2013). The communication between staff and TSs was not reported as being smooth in the literature either (Asikainen et al., 2018). The staff in our study reported that classes were the only settings in which they were able to interact with TSs, with very little opportunities to interact with TSs outside classes. Since previous studies have suggested that interactions between staff, particularly academic staff, and undergraduates outside classes and contact hours, can exert positive impacts on students’ academic and social adjustment to their university studies (Nel, 2014; Asikainen et al., 2018), it is recommended that academic staff maximize the potential of formal channels such as academic advising sessions and staff-student consultative meetings to identify the needs of individual TS and, if there is insufficient time and capacity to offer personalized assistance and to refer TSs to the resources and support available in the university (Smith and Allen, 2006; Nel, 2014; Ching et al., 2020a). Alternatively, informal channels such as social media (e.g., WhatsApp, Facebook) groups can be set up to prompt more spontaneous dialogues, both among TS communities and between academic staff, supporting staff, and students (Pramela et al., 2016). This kind of communication can be essential for encouraging TSs’ social involvement, especially when their heavy study loads discourage them from extra-curricular engagement and activities (McCormick et al., 2009). Such communication between staff and students can also allow staff to gain insights into the experiences of TSs outside formal contexts, so that they can be aware of or directly informed about the needs of TSs.

As one of the major findings in this study, the challenges encountered by TSs are acknowledged by the consequences of both limited credit transfer and the mandatory 2-year curriculum. As the common goals of timely degree attainment among the TSs, they need to finish the extensive study load in the limited timeframe and sacrifice their time in other interests, such as sports and social life (Ching et al., 2021). Furthermore, the staff in our study agreed that the extension of curricula would help TSs. An example of this kind of extension can be seen in the United States, in previous data reported by the National Student Clearinghouse Research Center (Shapiro et al., 2017), which indicated that about 9–14% of all 2-to 4-year TSs had not yet completed their bachelor’s degrees within 4 years of transferring and only half had completed their bachelor’s degrees within 6 years of starting at the community college. This implies that the predefined study durations for TSs might not be enough for them to complete bachelor’s degrees (Shapiro et al., 2018). Moreover, the impact of credit loss during the transfer process is also another factor causing delays to the degree completion of TSs (Monaghan and Attewell, 2015; Walker et al., 2016). These findings were consistent with our results that indicate the need for both the government and university management to review the current policies and practices related to the transfer pathway (e.g., credit transfer system) as well as to identify the needs of TSs during their university studies. A previous study also suggested that more credit transfers granted in the university could contribute to their success (Ishitani, 2008). Ishitani urged receiving institutions to evaluate their credit transfer process and granting mechanisms. For TSs, an organized cross-institutional credit transfer information online platform could enhance the ease of access to credit and program transfer information (Cheung et al., 2021), thus helping to minimize the credit loss and facilitate the study planning.

As said before, the university staff in this study expressed their sympathy about the difficulties TSs have to face, but have limited ability to help them due to the funding restrictions policy. Given that neither academic nor supporting staff are able to change the structure of the transfer pathway, such as lengthening the period of degree studies or alleviating their workloads, it still remains at their discretion to endeavor to offer *ad hoc* assistance and support for these “disadvantaged” non-traditional students (Zhang et al., 2018). It is necessary to raise awareness among different stakeholders in Hong Kong’s local higher education context about the benefits of the TSs. These stakeholders should include government funding and higher education governing sectors, and university and community college management, academic and supporting staff about the needs and challenges encountered by TSs. A cross-institutional and sectoral collaboration is suggested to inform all the stakeholders to discuss the current practices and benchmarks with overseas experiences for the sake of the local higher education context. Ultimately, concerted efforts should again be made by both academic departments and supporting units to collaborate at school level, to ensure a better structured and academically and socially supported transfer pathway for TS as a group of under-represented and underserved students. Furthermore, policy makers should not only emphasize improving the program alignment between community college and university, but also the learning environment to facilitate the seamless “2+2” articulation. This will maximize the resources spent on the 2-year community college and 2-year university study.

### Limitations

Our study had several limitations. First, reporting bias might have been present as some participants may not have expressed their opinions in full about some topics, such as university policies and management issues, due to their identity in spite of anonymity. Second, various departments might have different numbers of TSs, which would mean that interviewees might have varied extents of interactions with TSs that might have affected their experiences. Last, we acknowledge that one of the major limitations of this study is that the interviewees were only recruited from one university, albeit the one hosting the most TSs in Hong Kong {about 35% of the total TSs intake in recent 5 years [Concourse on Self-financing Post-secondary Education (CSPE), 2020]}. To yield more comprehensive perspectives, future work will investigate university staff’s perceptions of community college transfer pathways and TSs from other institutions in Hong Kong.

## Conclusion

In this study, we investigated university staff’s perceptions of TSs’ learning within the “2+2” pathway in an Asian context. The university staff showed confidence in the alternative pathway to university studies, but they also indicated major obstacles hindering them from supporting TSs in this “2+2” pathway. The staff agreed that the quality of TSs is aligned to the university standard as the entry requirement (i.e., strongest community college graduates) is maintained at a high level, and most TSs essentially have positive learning attitudes and outstanding academic performances. Likewise, these TSs value this second chance and are highly motivated to strive for timely baccalaureate degree completion despite the hardships in adjustment and learning process. However, the university staff observed that a negative consequence of the TSs’ effort is the pressure from their high self-expectations for academic achievement and to complete their studies within a fixed 2-year timeframe, thus losing the time and capacity to participate in social events and overseas exchange. Although the university staff recognized TSs’ challenges during the transition, they felt limited in resolving the situation as the 2+2 pathway is a consequence of funding and time restrictions. In order to facilitate TSs’ learning experiences, university staff could enhance the interactions with TSs outside classes to give them support. At the same time, both university and government managements should review the current policies and practices to facilitate the seamless “2+2” transfer pathway articulation.

## Data Availability Statement

The original contributions presented in the study are included in the article, further inquiries can be directed to the corresponding author.

## Ethics Statement

The studies involving human participants were reviewed and approved by the Human Subjects Ethics Sub-Committee, The Hong Kong Polytechnic University (HSEARS20180104005-01). The participants provided their written informed consent to participate in this study.

## Author Contributions

SC and KC: conceptualization and validation. WK and LZ: methodology. WK, JN, LZ, and CH: formal analysis. WK, LZ, and CH: data curation. WK, JN, and LZ: writing—original draft preparation. SC, WK, JN, CH, and KC: writing—review and editing. SC, WK, JN, LZ, CH, and KC: project administration. KC: funding acquisition. All authors have read and agreed to the published version of the manuscript.

## Conflict of Interest

The authors declare that the research was conducted in the absence of any commercial or financial relationships that could be construed as a potential conflict of interest.

## Publisher’s Note

All claims expressed in this article are solely those of the authors and do not necessarily represent those of their affiliated organizations, or those of the publisher, the editors and the reviewers. Any product that may be evaluated in this article, or claim that may be made by its manufacturer, is not guaranteed or endorsed by the publisher.
